# Emerging Technologies for Exploring the Cellular Mechanisms in Vascular Diseases

**DOI:** 10.3390/ijms27010164

**Published:** 2025-12-23

**Authors:** Debasis Sahu, Treena Ganguly, Avantika Mann, Yash Gupta, Logan R. Van Nynatten, Douglas D. Fraser

**Affiliations:** 1 Science Habitat, Ubioquitos Inc., 301-1554 Trossacks Ave, London, ON N5X 2P4, Canada; treenapganguly.66@gmail.com (T.G.); avantika.mann@gmail.com (A.M.); 2 Division of Gastroenterology and Hepatology, Department of Medicine, Penn State University College of Medicine, Hershey, PA 17033, USA; yashodharmangupta@gmail.com; 3 Division of Critical Care Medicine, Department of Medicine, Western University, London, ON N6A 5W9, Canada; logan.vannynatten@lhsc.on.ca; 4 Children’s Health Research Institute, London, ON N6C 2V5, Canada; 5 Department of Pediatrics, Western University, London, ON N6A 5C1, Canada; 6 Department of Physiology & Pharmacology, Western University, London, ON N6A 3K7, Canada; 7 Department of Clinical Neurological Sciences, Western University, London, ON N6A 3K7, Canada; 8 GSK Chair in Clinical Pharmacology, Western University, London, ON N6A 3K7, Canada; 9 Room A5-132, London Health Sciences Centre, 800 Commissioners Road East, London, ON N6A 5W9, Canada

**Keywords:** cardiovascular disease, omics, machine learning, systems biology, precision medicine, biomarkers

## Abstract

Vascular diseases (VDs) and cardiovascular diseases (CVDs) are the leading causes of morbidity and mortality worldwide. Current diagnostic and therapeutic approaches are limited by insufficient resolution and a lack of mechanistic understanding at the cellular level. Traditional imaging and clinical assays do not fully capture the dynamic molecular and structural complexities underlying vascular pathology. Recent technological innovations, including single-cell and spatial transcriptomics, super-resolution and photoacoustic imaging, microfluidic organ-on-chip platforms, Clustered Regularly Interspaced Short Palindromic Repeats (CRISPR)/CRISPR-associated protein 9 (Cas9)-based gene editing, and artificial intelligence (AI), have created new opportunities for investigating the cellular and molecular basis of VDs. These techniques enable high-resolution mapping of cellular heterogeneity and functional alterations, facilitating the integration of large-scale data for biomarker discovery, disease modeling, and therapeutic development. This review focuses on evaluating the translational readiness, limitations, and potential clinical applications of these emerging technologies. Understanding the cellular and molecular mechanisms of VDs is essential for developing targeted therapies and precise diagnostics. Integrating single-cell and multiomics approaches highlights disease-driving cell types and gene programs. Optogenetics and organ-on-chip platforms allow for controlled manipulation and physiologically relevant modeling, while AI enhances data integration, risk prediction, and clinical interpretability. Future efforts should prioritize multi-center, large-scale validation studies, harmonization of assay protocols, and integration with clinical datasets and human samples. Multi-omics approaches and computational modeling hold promise for unraveling disease complexity, while advances in regulatory science and digital simulation (such as digital twins) may further accelerate personalized medicine in vascular disease research and treatment.

## 1. Introduction

Vascular diseases (VDs) continue to be a major cause of morbidity and mortality worldwide, contributing significantly to the global burden of cardiovascular diseases (CVDs). According to the World Health Organization (WHO), approximately 18 million people die each year from CVDs, with ischemic heart disease and stroke being the leading contributors [1]. The underlying pathology of most CVDs is atherosclerosis, a chronic inflammatory and degenerative process of the vascular wall that leads to events such as myocardial infarction, stroke, aneurysm rupture, and peripheral arterial disease [2]. Established risk factors, including smoking, obesity, hyperlipidemia, hypertension, and advancing age, accelerate vascular injury and maladaptation [3,4]. Despite advances in surgery, endovascular procedures, and pharmacological strategies, treatment options remain limited, and outcomes are unsatisfactory for many patients [5]. Conventional diagnostic and research tools, such as angiography, ultrasound, computed tomography (CT), and magnetic resonance imaging (MRI), have provided invaluable insights into vascular pathology; however, they are limited by their resolution, sensitivity, and inability to capture dynamic molecular processes [6]. Addressing these limitations requires novel methodologies that interrogate vascular disease at the cellular and subcellular levels, offering mechanistic insights and translational opportunities [7,8,9].

In recent years, an array of new technologies has reshaped the study of vascular biology. Single-cell RNA sequencing (scRNA-seq) and spatial transcriptomics have enabled unprecedented resolution of cellular heterogeneity and tissue architecture in vascular lesions [10,11]. Microfluidic organ-on-chip platforms replicate physiological shear forces and multicellular interactions, bridging the gap between reductionist in vitro models and animal studies [11,12,13]. Optical and acoustic imaging approaches, including optogenetics, super-resolution microscopy, and photoacoustic tomography, provide spatiotemporal access to vascular dynamics [14]. At the genetic level, Clustered Regularly Interspaced Short Palindromic Repeats (CRISPR)/CRISPR-associated protein 9 (Cas9) or CRISPR/Cas9 enables precise perturbation of candidate pathways implicated in endothelial dysfunction and smooth muscle cell plasticity [15]. Computational frameworks, particularly machine learning (ML) and explainable artificial intelligence (AI), facilitate the integration of complex omics and imaging datasets for biomarker discovery, disease modeling, and risk prediction [16].

**Figure 1:**
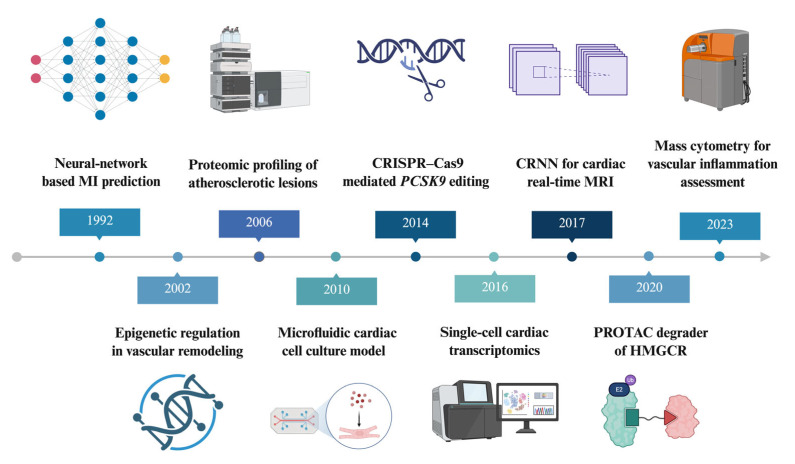
Timeline of technological emergence in cardiovascular research. The schematic illustrates selected milestones that underpin the technologies discussed in this review. Early artificial neural-network models for diagnosing myocardial infarction from clinical data (1992) [17]. In 2002, altered methylation of genomic DNA was found in human atherosclerotic lesions, implicating epigenetic regulation in vascular remodeling [18]. Proteomic mass spectrometry profiling of human atherosclerotic plaques was performed in 2006 [19]. Development of microfluidic cardiac cell culture models (µCCCM) was performed in 2010 [20]. In 2014, in vivo CRISPR-Cas9 editing of the *PCSK9* gene was performed to reduce the plasma cholesterol [21]. Single-cell temporal gene expression enabled the deciphering of cardiac development trajectories. Transcriptomic profiling was used to map anatomically patterned subpopulations within single embryonic cardiac cells [22]. The CRNN study helped to reconstruct highly undersampled dynamic cardiac MR images [23]. In 2020, PROTAC was used to target the degradation of HMG-CoA reductase, reducing cholesterol biosynthesis without HMGCR upregulation [24]. Mass cytometry profiling was performed to assess vascular inflammation [25]. Abbreviations: MS (mass spectrometry); CRISPR-Cas9 (Clustered Regularly Interspaced Short Palindromic Repeats-CRISPR-associated protein 9); CRNN (convolutional recurrent neural network); PROTAC (proteolysis targeting chimeras); HMGCR (3-Hydroxy-3-Methylglutaryl-CoA Reductase). The figure was created in BioRender. Sahu, D. (2025) https://www.biorender.com/ (accessed on 12 November 2025).

In this review, we focus on emerging technologies that provide cellular or near-cellular resolution and physiologically relevant modeling of vascular systems, and that have been applied to vascular or cardiovascular disease contexts or show a clear translational pathway. Specifically, we include single-cell and spatial omics, advanced imaging modalities, microfluidic and organ-on-chip systems, genetic perturbation tools such as CRISPR/Cas9 and optogenetics, and AI-based computational frameworks, including multiscale models and digital twins. Our central question is how these platforms deepen mechanistic understanding of vascular disease and how close each is to clinical translation, from discovery to early human applications. This review presents a timeline perspective on these emerging assays and platforms, highlighting their contributions to unraveling the cellular mechanisms underlying VDs. Figure 1 presents the evolutionary history of the techniques reviewed.

We discuss the key applications, strengths, limitations, and translational potential of each approach, emphasizing how technology integration can accelerate the development of clinically actionable biomarkers and therapies.

This study highlights emerging methodologies for understanding vascular disease mechanisms, emphasizing their historical development and translational potential.

### Literature Selection

We identified relevant studies through searches of PubMed and Web of Science using combinations of terms related to vascular disease (e.g., “atherosclerosis,” “aneurysm,” “pulmonary hypertension,” “peripheral artery disease”), emerging technologies (e.g., “single-cell RNA sequencing,” “spatial transcriptomics,” “photoacoustic imaging,” “organ-on-chip,” “digital twin”), and translational keywords (e.g., “biomarker,” “clinical trial,” “validation”). We prioritized human studies and clinically relevant animal or in vitro models that provided mechanistic insight or translational implications. When describing technologies, we emphasize applications that directly address vascular biology, rather than generic technical capabilities.

## 2. Cellular and Molecular Resolution Approaches

### 2.1. Single-Cell RNA Sequencing

Over the past decade, advances in scRNA-seq technologies have enabled transcriptomic profiling at the individual cell level. Numerous studies have demonstrated that scRNA-seq is valuable for exploring epigenetics and cellular heterogeneity in VDs [26,27,28]. Significant differences in DNA methylation and histone modification patterns have been observed between cells from healthy and diseased aortic tissues. Researchers have identified several differentially methylated or modified genes related to inflammation and smooth muscle cell function, highlighting considerable heterogeneity in epigenetic modifications within both healthy and diseased samples [29]. These epigenetic changes may occur in specific cell types or subpopulations, which could have implications for the development of targeted therapies for CVDs. Further studies have explored the role of epigenetics in vascular disease [30]. For example, notable differences in DNA methylation patterns between atherosclerotic and non-atherosclerotic arteries suggest that epigenetic changes may contribute to atherosclerosis. Studies of histone modifications in vascular calcification have demonstrated that these modifications regulate gene expression. Additionally, research on abdominal aortic aneurysms has shown substantial differences in DNA methylation patterns between aneurysmal and non-aneurysmal tissues. Investigations of pulmonary arterial hypertension have revealed how histone modifications can influence gene expression related to this condition [31,32,33].

**Figure 2:**
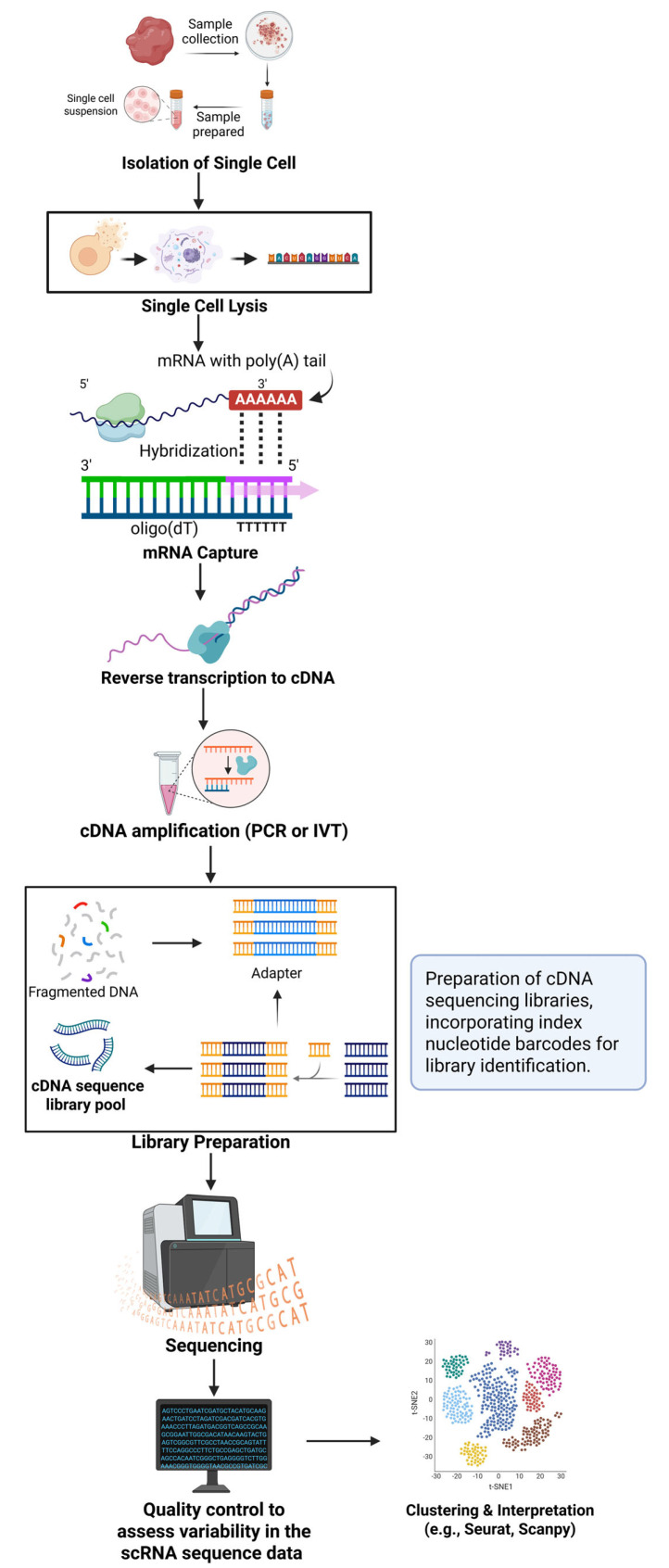
Workflow of single-cell RNA sequencing (scRNA-seq). The process begins with sample collection and preparation of a single-cell suspension, followed by isolation of individual cells. Each cell undergoes lysis to release mRNA, which is captured through hybridization with oligo(dT) primers targeting poly(A) tails. The captured mRNA is reverse-transcribed into cDNA, which is then amplified by PCR or IVT. Amplified cDNA is fragmented, and sequencing adapters with unique index barcodes are incorporated during library preparation. The prepared libraries are then sequenced to generate transcriptomic data. Subsequent quality control assesses variability and removes technical noise. Finally, bioinformatic tools such as Seurat or Scanpy are applied to cluster, visualize, and interpret cellular heterogeneity and gene expression patterns at single-cell resolution. Abbreviations: cDNA (complementary DNA); scRNA-seq (single-cell RNA sequencing); PCR (polymerase chain reaction); IVT (in vitro transcription). The figure was created in BioRender. Figure credit: Sahu, D. (2025) https://www.biorender.com/ (accessed on 12 November 2025).

Interactions between different cell types are crucial for the development and progression of VDs, and scRNA-seq can elucidate these interactions. For example, the interplay between endothelial cells and smooth muscle cells is essential for normal vascular function, and dysfunction in this interaction can lead to disease. The scRNA-seq technology enhances our understanding of the cellular mechanisms underlying these diseases by identifying involved cell types, gene expression patterns, and their interactions, providing new insights into pathogenesis. Identifying novel cell types and gene expression signatures is critical for developing targeted therapies that address specific cellular mechanisms in vascular diseases [34,35]. The first scRNA-seq study was published in 2009 [36]. This powerful technique enables the identification of previously unknown cell subtypes, whole-transcriptome profiling, elucidation of phenotypic transitions, and confirmation or expansion of knowledge about various organs and systems. scRNA-seq has now been successfully applied to multiple organs and organ systems [11,36]. Its general workflow includes cell isolation, mRNA extraction, cDNA synthesis, and sequencing (see Figure 2). An integration of scRNA-seq data from 44,120 immune cells in 17 human atherosclerosis samples identified key immune cell types, such as pro-inflammatory CD4+ CD28null T cells and dysfunctional TREM2-SPP1+ foamy macrophages, linked to atherosclerosis progression and poor outcomes, suggesting these as potential therapeutic targets for precision medicine [37]. Therefore, scRNA-seq can identify specific disease mechanisms and therapeutic targets that may improve patient outcomes. As sequencing costs continue to decrease, scRNA-seq analysis of millions of cells per study will become more accessible. The development of centralized data repositories will further facilitate access to valuable cellular-level information. Improvements in mRNA capture efficiency are also anticipated, enhancing the sensitivity of gene expression analyses [38,39,40,41]. Integrating scRNA-seq with other modalities, such as proteomics and epigenomics, provides a holistic view of cellular biology. Sc RNA-seq is thus a vital diagnostic tool, aiding the detection of rare cells and informing treatment decisions across various diseases. Based on the results of genome-wide association studies (GWAS) and single-cell transcriptomics of vascular disease, specific genes and pathways implicated in VSMC and fibroblast cell transitions have been identified. Most of these single-cell studies are based on relatively small cohorts or preclinical models, and many of the identified cell states and gene signatures remain candidate biomarkers that require validation in larger, independent vascular datasets.

Using scRNA-seq, researchers were able to detect a correlation between vascular inflammation symptoms in Kawasaki disease and those associated with B-lymphocytes [42,43]. Transcriptomic profiling via scRNA-seq identified three palmitoylated genes, suggesting a role for S-palmitoylation in coronary artery disease (CAD) [38]. Bioinformatics analysis of the CMD rat model, based on scRNA-seq findings, indicated reductions in endothelial cells, an increase in fibroblast cells, oxidative stress, and an inflammatory response. Essential transcription factor-1 (ETF-1) has been identified as a primary target for vascular endothelial injury in diabetic cardiac disease using scRNA-seq [44,45]. A transcriptomic study employing clinical samples from patients with pulmonary arterial hypertension (PAH) identified primary glycolysis genes (*CASP3*, *IGF1*, *CDKN2A*, and *KARS*) associated with PAH through combined analysis of scRNA-seq and bulk transcriptomic data [46]. Two potential biomarkers (COL1A1 and C1QC) were identified for their association with atherosclerosis using scRNA-seq in combination with machine learning [47]. Enhanced expression of circular RNA during vascular cell differentiation has also been reported, suggesting it may serve as a potential biomarker for VD [48].

### 2.2. Epigenetic and Chromatin Accessibility Methods

Beyond transcriptional profiling, epigenetic mechanisms such as DNA methylation and histone modifications critically regulate gene expression programs in vascular cells. Epigenetic remodeling in endothelial cells, vascular smooth muscle cells (VSMCs), and infiltrating immune cells has been linked to the development and progression of atherosclerotic plaques, aneurysm formation, and vascular remodeling [49]. For example, altered promoter methylation and histone acetylation patterns in genes controlling inflammation, matrix remodeling, and contractile function have been associated with unstable plaque phenotypes and maladaptive VSMC transitions [50].

Recent advances in single-cell chromatin accessibility profiling, such as single-cell transposase-accessible chromatin using sequencing (scATAC-Seq) assay, allow the mapping of open chromatin regions at the level of individual vascular cells [51]. This provides complementary information to scRNA-seq by identifying enhancers and promoters that are active in specific cell states, including disease-associated macrophages, modulated VSMCs, and activated endothelial cells. In vascular disease models, integrating chromatin accessibility with gene expression has begun to reveal regulatory elements and transcription factors that drive pathogenic phenotypes. However, single-cell epigenomic assays remain technically demanding, and most applications in vascular biology are still in the discovery phase, with limited multi-center validation.

### 2.3. Proteomic and Single-Cell Proteomic Approaches in Vascular Disease

Whereas transcriptomic and epigenomic assays capture regulatory potential, proteomic technologies provide a direct readout of the effector layer that executes cellular programs in vascular disease [52]. Mass spectrometry–based proteomics enables unbiased quantification of thousands of proteins within vascular tissues, circulating blood, or isolated cell populations, revealing pathways involved in inflammation, extracellular matrix remodeling, thrombosis, and metabolic stress [53]. In atherosclerotic plaques, aneurysmal walls, and pulmonary vascular lesions, proteomic profiling has highlighted dysregulated complement components, matrix metalloproteinases, coagulation factors, and adhesion molecules that may contribute to plaque instability, aneurysm rupture, or microvascular dysfunction [54].

Targeted and discovery proteomics have also been applied to plasma and serum, where they support the identification of candidate circulating biomarkers for VDs, including proteins linked to endothelial injury, smooth muscle cell phenotypic switching, and immune activation [55]. These circulating signatures can complement imaging and clinical risk scores and may help stratify patients according to inflammatory burden or vascular remodeling activity [56]. In parallel, advances in high-dimensional single-cell proteomic platforms, such as mass cytometry (CyTOF), imaging mass cytometry, and oligo-tagged antibody panels integrated with scRNA-seq (e.g., CITE-seq), permit simultaneous measurement of dozens to hundreds of protein epitopes per cell. In vascular research, these approaches enable fine-grained mapping of endothelial, vascular smooth muscle, fibroblast, and immune cell phenotypes, including activation states and receptor–ligand expression profiles that are not fully captured at the transcript level.

Metabolomic profiling, often combined with proteomic data, further refines our understanding of vascular pathophysiology by characterizing bioactive lipids, amino acid derivatives, and energy metabolites associated with endothelial dysfunction, oxidative stress, and vascular inflammation [57]. However, most proteomic and metabolomic studies in vascular disease still involve modest cohort sizes, heterogeneous sample processing, and variable analytical pipelines. As a result, many proposed protein and metabolite signatures remain at the discovery or early validation stage, with limited multi-center replication and uncertain incremental value over established clinical risk factors.

### 2.4. Strategies and Challenges of Multi-Omics Integration

It is increasingly clear that single-layer molecular analyses are insufficient to capture the complexity of vascular remodeling, calcification, and inflammatory activation [58]. Multi-omics strategies that combine single-cell RNA sequencing (scRNA-seq), single-cell chromatin accessibility profiling (e.g., ATAC-seq), and proteomic or metabolomic readouts offer a more holistic view of cellular states and regulatory networks in vascular disease [59]. By jointly interrogating gene expression, chromatin accessibility, and protein abundance, these approaches enable the reconstruction of gene regulatory circuits, the identification of disease-associated transcription factors, and the linkage of upstream regulatory changes to downstream effector pathways within specific endothelial, smooth muscle, fibroblast, or immune cell subsets [60].

In atherosclerosis, aneurysm formation, and pulmonary vascular remodeling, multi-omics integration has been used to refine cell-state taxonomies and to dissect transitions such as vascular smooth muscle cell (VSMC) phenotypic switching, endothelial–mesenchymal transition, and macrophage polarization [61]. For example, pairing scRNA-seq with scATAC-seq allows assignment of active enhancers and promoters to lineage-defining transcription factors in modulated VSMCs or lesional macrophages, while proteomic measurements validate whether predicted signaling pathways are implemented at the protein level [61]. Such “vertical” integration across transcriptomic, epigenomic, and proteomic layers increases confidence that identified pathways are not artifacts of a single assay and supports the nomination of more robust therapeutic targets.

Advances in computational pipelines have improved the feasibility of these studies by enabling alignment and joint analysis of heterogeneous data types from the same or closely matched cell populations [62]. Tools such as Seurat, Signac, and Harmony support multi-modal integration, allowing researchers to embed cells in shared low-dimensional spaces, infer gene regulatory networks, reconstruct cell-fate trajectories, and model intercellular communication within vascular lesions. Network-based and machine-learning approaches further prioritize features that are conserved across modalities and cohorts and can begin to link multi-omics signatures to imaging phenotypes or clinical outcomes [63].

Despite this promise, multi-omics integration in vascular disease faces substantial challenges. Tissue availability is often limited, especially for human vascular specimens, leading to small sample sizes and reduced statistical power. Differences in tissue handling, library preparation, and platform chemistry introduce batch effects that complicate cross-study comparisons. At the analytic level, issues such as data sparsity, modality-specific noise, missing features across assays, and the risk of overfitting high-dimensional datasets remain significant obstacles. Furthermore, most published multi-omics studies in vascular disease rely on retrospective or cross-sectional cohorts, with only preliminary evidence that integrated molecular signatures improve prediction beyond clinical and imaging variables [64]. Addressing these limitations will require standardized protocols, larger multi-center datasets, transparent reporting of integration workflows, and prospective validation of multi-omics-derived biomarkers and targets in independent vascular cohorts.

## 3. Experimental and Engineering Platforms

### Optogenetics

**Figure 3:**
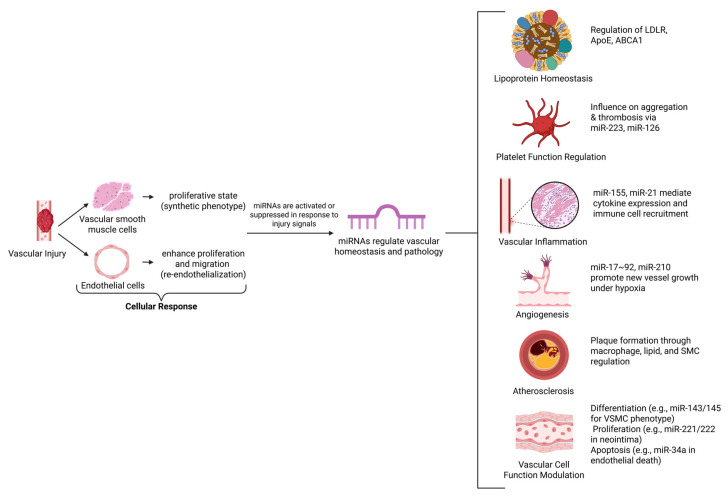
Role of microRNAs (miRNAs) in vascular injury response and cardiovascular pathology. Vascular injury triggers cellular responses in VSMCs and endothelial cells. VSMCs shift toward a proliferative synthetic phenotype, while endothelial cells enhance proliferation and migration during re-endothelialization. In response to these injury signals, miRNAs are activated or suppressed, thereby regulating vascular homeostasis and disease progression. Key processes influenced by miRNAs include lipoprotein homeostasis (e.g., regulation of *LDLR*, *ApoE*, *ABCA1*), platelet function and thrombosis (e.g., miR-223, miR-126), vascular inflammation (e.g., miR-155, miR-21), angiogenesis under hypoxia (e.g., miR-17~92, miR-210), atherosclerosis via lipid, macrophage, and smooth muscle regulation, and modulation of vascular cell functions such as VSMC differentiation (miR-143/145), proliferation (miR-221/222), and apoptosis (miR-34a). It highlights the multifaceted role of miRNAs in orchestrating vascular homeostasis, remodeling, and pathology. Abbreviations: miRNA (micro RNA); VSMC (vascular smooth muscle cells). The figure was created in BioRender. Figure credit: Sahu, D. (2025) https://www.biorender.com/ (accessed on 12 November 2025).

Optogenetics is a technique for controlling cellular activity using light, enabling precise manipulation of specific cell populations within heterogeneous tissues through genetic engineering. In recent years, optogenetics has become increasingly popular among researchers, enabling fine control over the expression of light-sensitive proteins. It has been used to investigate cell signaling, gene expression, and cell migration [65,66,67]. Optogenetics has been applied to study vascular smooth muscle cells (VSMCs), which undergo contractility changes that contribute to atherosclerosis in vascular disease [61,68]. Given the central role of VSMCs in VDs, optogenetics is now used to modulate their activity, providing new insights into underlying mechanisms. Optogenetic approaches control VSMC contractility and explore cellular mechanisms involved in VDs [69,70]. This technology has greatly enhanced our ability to study disease processes with unparalleled precision, revealing complex interactions between different cell types (Figure 3). Endothelium-targeted optostimulation has shown promise as an intervention for the vascular system. Optogenetics uses opsins to modulate cellular activity in response to light. Channelrhodopsins (e.g., ChR2), activated by 470 nm blue light, depolarize cells via sodium and calcium influx, enabling activation on the millisecond scale. Inhibitory opsins, such as halorhodopsins (NpHR, 590 nm yellow light) and archaerhodopsins (ArchT, 530 nm green light), hyperpolarize cells by pumping chloride or protons, effectively silencing activity [71]. Red-shifted variants like ChrimsonR (590 nm) enhance tissue penetration for deeper vascular targeting. Cell-type specificity is achieved using promoters (e.g., Myh11 for VSMCs, Tie2 for endothelial cells) delivered via adeno-associated viruses (AAVs) or lentiviral vectors, with Cre-lox systems allowing temporal control [72,73,74]. Light can be delivered by fiber optics or two-photon microscopy, providing 10–100 µm resolution, as demonstrated by 473 nm laser-induced calcium transients in murine carotid arteries [75].

While optogenetic approaches have yielded precise control of vascular and cardiac activity in preclinical models, including modulation of vascular tone and cardiac sympathetic nerve activity, no routine clinical applications in human vascular disease currently exist [76]. Major translational barriers include challenges in delivering light to deep vascular structures, safe and efficient gene delivery of opsins, potential immunogenicity, and the need for long-term safety data [77]. Consequently, most proposed therapeutic applications of optogenetics in vascular medicine remain speculative extensions of the experimental work described above.

Optogenetic regulation of cardiac sympathetic nerve (CSN) activity effectively prevents myocardial ischemia (MI)-induced ventricular arrhythmias [76]. However, optogenetics faces limitations, notably the requirement for light to activate light-sensitive proteins, which is a significant obstacle in vivo since light cannot always penetrate deep tissues [78]. A clinically applicable version of optogenetics remains under development. Combining optogenetics with systems biology approaches (e.g., transcriptomics, proteomics) helps elucidate network-level responses in cardiovascular disease (CVD). Organ-on-a-chip platforms integrated with optogenetics mimic vascular physiology and enable studies of mechanical stress or drug responses in human-relevant models [79]. These approaches enhance the translational relevance of optogenetic findings. The specific applications of optogenetics across various cardiovascular cell types and disease models, along with their associated mechanisms, challenges, and proposed future directions, are summarized in Table 1.

## 4. Imaging Innovations

Modern imaging technologies are transforming the study of vascular biology by extending the limits of resolution, penetration depth, and temporal fidelity beyond those of conventional modalities such as ultrasound, CT, and MRI. These advances have enabled visualization of vascular processes at scales ranging from nanometers to intact tissue, providing mechanistic insights previously inaccessible.

### 4.1. Super-Resolution Microscopy/Nanoscopy

Conventional imaging techniques such as X-rays, CT, and MRI have limitations in resolution and sensitivity. Recent advances in imaging techniques have enabled researchers to study VDs at the cellular level, offering insights into their mechanisms [80]. Super-resolution microscopy or nanoscopy techniques, including super-resolution ultrasound (SRU), multimodal ultrafast sonography microscopy (MUSM), ultrasound localization microscopy (ULM), photoacoustic tomography (PAT), intravital microscopy (IVM), SRμT (synchrotron radiation micro-tomography), and E-uPIV (enhanced ultrasound particle image velocimetry), allow visualization of intracellular structures beyond the diffraction limit of light [81]. In VDs, the breakdown of the endothelial glycocalyx compromises vascular permeability, increasing leukocyte access to the arterial intima, promoting inflammation, and disrupting protective endothelial signaling. Advanced imaging methods, such as super-resolution microscopy and intravital microscopy, provide real-time, high-resolution visualization of cellular processes, including leukocyte-endothelial interactions and platelet activation, in vascular disease.

Optical coherence tomography also provides detailed imaging of the arterial wall, aiding in assessing atherosclerosis progression and the effectiveness of interventions such as stenting. These technologies improve our understanding of vascular pathology and support the development of innovative diagnostic and therapeutic strategies [82]. Photoacoustic imaging, a noninvasive super-resolution microscopy technique, enables visualization of blood vessels and tissues with high resolution and sensitivity. Microvascular imaging of tumors and other diseases has been achieved using photoacoustic tomography [83]. In another study, photoacoustic imaging was used to assess the effects of high-fat diets on atherosclerosis development [84,85]. A recent investigation demonstrated that a high-fidelity 3D-PAT scanner is a promising tool for obtaining in vivo 3D images of vascular anatomy. High-resolution PAT enables visualization of deep-seated blood vessels across different body positions [86]. Three-dimensional photoacoustic imaging visualizes foot vasculature using multi-element (~128-element) transducers. SRμT refers to super-resolution microscopy techniques using synchrotron radiation to generate high-resolution 3D images beyond the diffraction limit [87]. SRμT provided high-resolution images of vascular calcification that were not achievable with other imaging methods.

A study using a murine model of Alzheimer’s disease detected microvascular dysfunction (MVD) with IVM, suggesting a role for MVD in the progression of Alzheimer’s disease pathology. Another investigation developed an intravital heart micro-imaging protocol enabling real-time imaging of cardiac tissue in a live animal model. Super-resolution ULM detects changes in blood flow by visualizing deep-seated restricted vessels. Human blood vessels can be resolved 5.7 times better with super-resolution ULM compared to contrast-enhanced power Doppler [86]. SRU facilitates visualization of vascular reorganization in the neonatal brain at depths of a few microns [88]. It also enables measurement of vascular flow dynamics and offers a sevenfold improvement in vascular imaging over traditional contrast ultrasound [89,90]. SRU is a reliable tool for obtaining high-resolution vascular images, including spatiotemporal images. In the L-NAME (Nω-nitro-L-arginine methyl ester) and angiotensin-II-induced hypertension mouse model, MUSM provides high-resolution images of vascular dynamics. E-uPIV allows visualization of microvasculature and accurate measurement of blood flow [91].

Intravital microscopy (IVM) adds a dynamic dimension by enabling direct visualization of the vascular microenvironment in living organisms. This technique allows real-time tracking of leukocyte–endothelial interactions, platelet adhesion, and thrombus formation under physiological shear forces. Intravital imaging has been indispensable for dissecting the spatiotemporal organization of inflammation, coagulation, and vascular remodeling, providing insights that cannot be gained from static tissue sections. Its versatility spans multiple organ systems, including the brain, heart, and peripheral vasculature, making it a powerful tool for studying how systemic diseases manifest at the microvascular level [92,93].

These imaging modalities complement one another; super-resolution microscopy excels at subcellular resolution, PAT offers label-free molecular contrast at tissue depth, and IVM reveals dynamic interactions in real time [94,95]. Together, they form a powerful imaging toolbox that links molecular events to functional outcomes in vascular disease. The translational trajectory is already emerging, especially with PAT, which is being piloted in clinical trials for vascular diagnostics. However, several challenges remain, such as the need for standardized protocols, miniaturization of imaging hardware, cost reduction, and robust validation across diverse patient populations.

### 4.2. Photoacoustic Imaging and Tomography

Photoacoustic imaging (PAI), including multispectral optoacoustic tomography (MSOT) and volumetric photoacoustic tomography, combines optical contrast with ultrasonic detection to enable high-resolution visualization of hemoglobin, lipids, and other chromophores in deep tissues. In preclinical vascular disease models and early human studies, PAI has been used to assess peripheral perfusion, microvascular remodeling, and plaque composition, providing functional information that complements Doppler ultrasound and conventional angiography. By capturing dynamic changes in tissue oxygenation and vascular density, photoacoustic techniques offer mechanistic insight into ischemia, neovascularization, and microvascular dysfunction. Recent clinical translation studies demonstrate that MSOT can discriminate symptomatic peripheral arterial disease (PAD) from healthy volunteers using hemoglobin-related calf-muscle biomarkers, while new all-optical three-dimensional PAI scanners deliver clinically useful microvascular images with acquisition speeds suitable for pilot clinical evaluations. Collectively, these advances highlight PAI’s potential as a bedside adjunct to Doppler ultrasound and angiography for functional perfusion assessment and plaque microenvironment phenotyping in PAD, diabetic microangiopathy, and related vascular disorders [96].

## 5. Microfluidic and Organ-on-Chip Systems

### 5.1. Hemodynamic Modeling

Microfluidic technologies and organ-on-chip (OoC) systems have emerged as powerful experimental platforms for modeling vascular biology in controlled environments that recapitulate key physiological conditions. Unlike conventional static culture systems, these devices utilize microscale channels lined with endothelial or smooth muscle cells. They are perfused with fluid to mimic the mechanical and biochemical cues experienced in vivo. This approach enables precise investigation of how hemodynamic forces, cellular interactions, and biochemical gradients contribute to vascular health and disease [94,97,98].

One of the most significant advantages of these systems is their ability to model hemodynamics and shear stress. Endothelial cells cultured under laminar flow conditions in microfluidic channels align in the direction of flow and exhibit anti-inflammatory, antithrombotic phenotypes, closely resembling the behavior of vascular endothelium in vivo [99]. In contrast, disturbed flow patterns, such as those at arterial bifurcations, can be replicated in microfluidic devices and have been shown to promote endothelial dysfunction, oxidative stress, and pro-atherogenic signaling [100]. These models enable researchers to dissect mechanotransduction pathways and test how pharmacological interventions might restore protective endothelial responses.

### 5.2. Thrombosis-on-Chip and Patient-Derived Platforms

Microfluidic platforms have also advanced thrombosis research. “Thrombosis-on-chip” devices simulate blood flow across surfaces coated with collagen or tissue factor, enabling real-time visualization of platelet adhesion, aggregation, and fibrin formation [101]. These systems have proven invaluable for studying the mechanisms of clot initiation, evaluating patient-specific coagulation profiles, and screening novel antithrombotic agents under physiologically relevant shear conditions. By replicating arterial or venous flow rates, these chips enable a more accurate assessment of thrombosis risk than static assays.

Another innovation is the incorporation of patient-derived induced pluripotent stem cells (iPSCs) into vascular chips. These iPSC-derived endothelial or smooth muscle cells allow modeling of genetic vascular disorders, such as aneurysm syndromes or inherited thrombophilias, in a personalized context. Using cells from affected individuals, OoC platforms can replicate disease-specific phenotypes and serve as testbeds for evaluating targeted therapeutic interventions. This personalized approach offers exciting opportunities for precision medicine, where therapies can be tailored and tested in vitro before clinical application [102,103,104].

Despite their promise, microfluidic and OoC technologies face important limitations. Variability in device design and fabrication complicates reproducibility across laboratories. Throughput remains limited compared to high-content screening methods, and standardization of culture conditions, readouts, and validation criteria is still evolving. Furthermore, while these systems approximate vascular physiology more closely than static cultures, they cannot fully replicate the complexity of whole-organ or systemic interactions. Nevertheless, these platforms have considerable potential. By bridging the gap between reductionist cell culture and animal models, microfluidic and organ-on-chip technologies provide a versatile and physiologically relevant environment for vascular research. As engineering approaches mature and protocols become standardized, these systems will likely play an increasingly central role in drug discovery, disease modeling, and personalized medicine for VDs.

### 5.3. Vascular Organoids and Organ-on-a-Chip Systems

Organ-on-a-chip platforms enhance physiological relevance by integrating microfluidic channels and controlled biomechanical cues (e.g., shear stress, pulsatile flow) into engineered vascular constructs. These systems mimic organ-level functions and vascular microenvironments, allowing real-time observation and manipulation of cell behavior under near-physiological conditions [105,106]. For example, models simulating blood–brain barrier (BBB) permeability have been developed to study vascular leakage in stroke and neurodegenerative conditions [107]. Vascular chips that mimic hypertensive microcirculation enable the investigation of endothelial barrier disruption and remodeling under elevated pressure gradients. These organ-on-a-chip systems allow modeling of disease-specific phenotypes, including inflammatory endothelial activation, thrombogenesis, and oxidative stress. Additionally, these technologies are highly amenable to high-throughput drug screening and personalized medicine, as they can incorporate patient-derived induced pluripotent stem cells (iPscRNA-seq) to generate genetically matched vascular tissues for therapeutic testing [108].

Importantly, vascular organoids and chips support multicellular integration, enabling the study of complex interactions among endothelial cells, vascular smooth muscle cells, immune cells, and fibroblasts. These cellular interactions are critical in processes such as atherosclerosis, restenosis, and vascular calcification. Furthermore, these platforms provide a controlled environment for assessing the effects of therapeutic agents on vascular permeability, tone regulation, and inflammatory responses, making them valuable tools for preclinical drug development and toxicology assessment.

### 5.4. Limitations, Reproducibility, and Ethical Considerations

Despite their promise, microfluidic and organ-on-chip technologies face essential limitations. Device design, fabrication protocols, and surface treatments vary across laboratories, leading to substantial variability in readouts and complicating reproducibility. Endothelial cells and supporting stromal cells exhibit marked heterogeneity across vascular beds, donors, and culture conditions, and capturing this diversity in simplified chip systems remains challenging. Long-term culture under flow is technically demanding, and maintaining stable, physiologically relevant phenotypes over extended periods is difficult. Moreover, current platforms often lack the full systemic context of human vascular disease, including interactions with the immune, nervous, and endocrine systems. The use of patient-derived iPSCs raises logistical and ethical questions related to consent, data protection, and equitable access to these personalized models. As a result, vascular microfluidic and organ-on-chip systems should be viewed as intermediate models that complement, rather than fully replace, animal studies and clinical investigations.

## 6. Computational and Genetic Approaches

### 6.1. CRISPR/Cas9 Gene Editing

**Figure 4:**
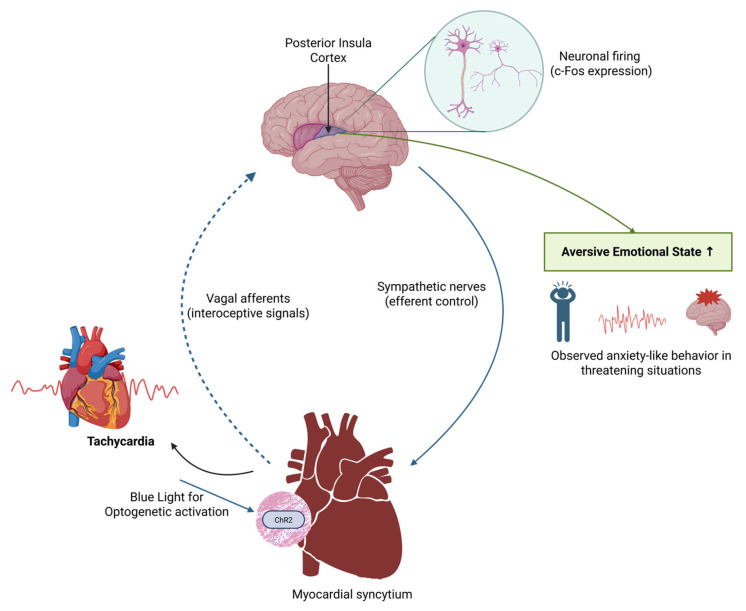
Optogenetic modulation of cardiac activity and its impact on emotional state. Blue light activation of channelrhodopsin-2 (ChR2) in the myocardial syncytium induces tachycardia, which sends interoceptive signals through vagal afferents to the posterior insula cortex. This activation promotes neuronal firing (c-Fos expression) and sympathetic efferent responses, linking altered cardiac rhythms to central nervous system processing. The resulting feedback contributes to an aversive emotional state, manifested as anxiety-like behavior in threatening situations. This framework illustrates how optogenetic cardiac stimulation can be used to probe heart–brain interactions underlying emotion regulation. Abbreviations: ChR2 (channelrhodopsin-2). The figure was created in BioRender. Sahu, D. (2025) https://www.biorender.com/ (accessed on 12 November 2025).

CRISPR/Cas9 is a novel technology that utilizes cut-and-paste mechanisms to enable gene modifications. This approach provides a better understanding of underlying disease mechanisms and opens new avenues for developing treatments [109,110]. Evidence from investigations has shown that CRISPR/Cas9 is a useful tool for studying CVDs. The involvement of the ATP-binding cassette transporter (*ABC*)*A1* gene in atherosclerosis development has been documented using CRISPR/Cas9 technology. Various microRNAs implicated in the development of atherosclerosis and hypertension include miR-126, miR-155, and miR-21 (Figure 4). MiR-126 is an endothelial-specific microRNA that plays a critical role in vascular homeostasis, and its deletion using CRISPR/Cas9 leads to impaired angiogenesis and increased susceptibility to atherosclerosis [111]. In a CRISPR/Cas9 gene editing study on mouse models, miR-145 ablation increased atherosclerotic plaque formation. One study demonstrated that deletion of miR-33 led to reduced atherosclerosis and improved lipid metabolism. Another study designed a CRISPR/Cas9 gene editing model to analyze the functional aspects of missense variants identified in CHD [112]. Additionally, a CRISPR/Cas9 study found that Cas9 expression did not impact cardiac function [113]. Several studies have examined the role of various genes and proteins in the development mechanisms of genetic heart disease.

These underscore the precision of single-cell approaches in identifying functional genetic elements. But to date, most CRISPR/Cas9 applications in vascular biology have been confined to in vitro systems or animal models, providing mechanistic insights into gene function and disease pathways. No CRISPR-based therapies are yet approved specifically for vascular indications, and major challenges remain regarding off-target effects, safe and efficient delivery to vascular tissues, immune responses, and long-term safety assessment.

The key molecular targets, including specific genes and microRNAs explored in cardiovascular disease using functional studies and genome-editing approaches, are further summarized in Table 2.

### 6.2. Machine Learning (ML) and Explainable AI (XAI)

While machine learning (ML) and artificial intelligence (AI) have shown substantial promise in predicting CVD risk, patient outcomes, and treatment response, a significant limitation of traditional models is their “black-box” nature. These models often lack interpretability, making it difficult for clinicians to understand the rationale behind predictions. Explainable artificial intelligence (XAI) addresses this challenge by providing tools and frameworks that elucidate the decision-making process of AI models, thereby enhancing their transparency, interpretability, and clinical trust, In the vascular domain, ML models have been developed to automatically segment vessels, quantify plaque burden, and predict outcomes such as stroke or limb ischemia from CT angiography, MRI, and ultrasound data. XAI approaches, including feature attribution methods, have begun to reveal which imaging-derived features and clinical variables most strongly drive these predictions, providing potential mechanistic clues and highlighting candidate biomarkers.

XAI techniques allow researchers and clinicians to identify key features, such as specific genes, signaling pathways, imaging-derived variables, or clinical biomarkers, that most significantly contribute to model predictions. For instance, when applied to large-scale patient data in vascular disease contexts, XAI can reveal which molecular signatures (e.g., pro-inflammatory cytokines or extracellular matrix-related genes) are most associated with plaque instability or vascular remodeling [114,115]. These insights go beyond mere classification, providing mechanistic clues into disease progression and patient heterogeneity.

The application of XAI in cardiovascular research is a critical advancement for bridging the gap between computational model outputs and actionable clinical knowledge. XAI frameworks, such as Shapley Additive exPlanations (SHAP), Local Interpretable Model-agnostic Explanations (LIME), and counterfactual reasoning, can be applied to diverse data modalities, including transcriptomics, imaging, and electronic health records. For VDs specifically, these tools facilitate the dissection of complex, nonlinear relationships between risk factors (e.g., hypertension, lipid levels, genetic variants) and disease phenotypes, such as arterial stiffness, aneurysm formation, or calcific lesion burden.

Importantly, XAI improves model transparency and supports the development of clinically actionable models. Identifying influential features facilitates risk stratification, prioritization of therapeutic targets, and refinement of personalized treatment strategies. For example, if an AI model predicts a high risk for atherosclerotic events, XAI can specify whether the prediction was driven primarily by inflammatory gene expression, vascular imaging patterns, or metabolic profiles, enabling more precise interventions. Furthermore, XAI can help validate novel hypotheses derived from omics data in translational research settings. When integrated with multi-omics approaches, XAI can trace how gene regulatory changes translate into phenotypic outcomes and link them with clinically measurable features, such as ankle-brachial index or carotid intima-media thickness. This cross-validation improves both model robustness and biological relevance.

A persistent challenge is that many models are trained and validated on single-center datasets with limited demographic diversity, which raises concerns about generalizability and bias. Performance can deteriorate when models are applied to new scanners, imaging protocols, or patient populations. Robust external validation, prospective testing, and monitoring of failure modes are therefore essential before ML and XAI tools can be safely integrated into routine vascular care.

### 6.3. Generative AI Models: VAEs and GANs

Generative artificial intelligence (AI) models, particularly variational autoencoders (VAEs) and generative adversarial networks (GANs), represent a powerful advancement in deep learning and are increasingly applied in CVD research. Unlike discriminative models, which focus solely on classification or regression, generative models learn the underlying data distribution, enabling them to generate new synthetic data that closely mimics real-world biological variation. These capabilities are leveraged to simulate disease progression, augment limited datasets, and support virtual experimentation in vascular biology.

Variational autoencoders (VAEs) combine Bayesian inference principles with neural encoding-decoding architectures to learn compressed, low-dimensional representations of complex datasets. In vascular research, VAEs can be applied to high-dimensional omics or imaging data to identify latent variables that describe the progression from healthy vasculature to pathological states such as atherosclerosis, arterial stiffness, or aneurysm formation. These latent variables can serve as interpretable biomarkers or be used to simulate disease trajectories under different genetic or environmental conditions [116,117,118]. For example, VAEs trained on time-series omics data can model smooth transitions in cell states during vascular remodeling or endothelial-to-mesenchymal transition.

GANs consist of a generator and a discriminator network trained in a competitive framework, making them particularly adept at producing high-fidelity synthetic data. In the cardiovascular domain, GANs have been employed to generate synthetic vascular images (e.g., CT angiography, ultrasound, histopathology) that preserve the structural and pathological features of real data [119,120,121]. This approach is especially valuable when annotated clinical datasets are scarce or imbalanced. GAN-generated data can augment training sets for downstream AI tasks such as vessel segmentation, plaque detection, or phenotype classification, thereby improving model robustness and generalizability.

Advanced applications of GANs also include disease progression modeling, in which the generator is conditioned to simulate vascular changes over time, such as calcification accumulation, lumen narrowing, or wall thickening under various risk scenarios. Conditional GANs (cGANs) and style-transfer GANs can further transform healthy tissue images into synthetic diseased states, providing insights into early pathological changes that may not be apparent in limited clinical samples [122,123].

Furthermore, generative models can assist in in silico experimental design, allowing simulated vascular datasets to be used for hypothesis testing, treatment response prediction, and modeling patient-specific disease risk. Integrating multi-omics and imaging data enables these models to generate biologically plausible outputs that reflect the interplay between gene regulation, protein expression, and structural remodeling. While generative AI models offer substantial promise, challenges remain, including ensuring biological plausibility, avoiding mode collapse in GANs, and validating synthetic data against empirical findings. As training frameworks, interpretability tools, and validation protocols advance, generative models are poised to become integral to predictive vascular research and precision cardiovascular medicine. The technologies ranging from single-cell omics and advanced imaging to organ-on-chip platforms and CRISPR/Cas9 systems represent a profound shift in vascular research. While each offers unique strengths in resolution or physiological relevance, they all face shared and distinct limitations concerning scalability, cost, in vivo applicability, and regulatory approval. To provide a comparative assessment and set the stage for discussing multimodal integration, Table 3 offers a synthesis of the key features, applications, strengths, limitations, and overall translational potential of the emerging techniques.

### 6.4. Multiscale Modeling: Linking Physics, AI, and Omics

Multiscale modeling integrates mathematical and computational approaches that span different biological and physical scales, from molecular dynamics and gene regulatory networks to cell–cell interactions, tissue biomechanics, and whole-organ hemodynamics [124]. Traditionally, computational fluid dynamics (CFD) models have been used to study vascular flow patterns, shear stress, and pressure distributions in arteries. Recent advances connect these physics-based models with ML algorithms and omics data, enabling a more comprehensive understanding of how molecular changes manifest as measurable hemodynamic outcomes [125,126].

CFD models simulate blood flow using the Navier–Stokes equations, applied to patient-specific anatomical geometries derived from CT or MRI angiography. When integrated with omics data (such as endothelial gene expression profiles under disturbed flow), these models can predict how shear stress influences inflammatory activation, plaque formation, or endothelial dysfunction [127]. ML and AI are increasingly used to optimize model parameters, reduce computational burden, and uncover nonlinear relationships between local flow environments and disease phenotypes.

Despite significant advances, multiscale models are still limited by high computational costs, complex boundary condition requirements, and uncertainties in integrating molecular data with macroscopic hemodynamics [28,128]. Model validation remains challenging because it is difficult to obtain simultaneous ground-truth hemodynamic and molecular measurements in humans. Furthermore, interoperability among datasets, imaging, omics, and biomechanical models is not yet standardized, which hampers reproducibility across studies.

### 6.5. Digital Twins: Toward Personalized Vascular Simulations

A digital twin is a virtual replica of a patient’s vascular system that is continuously updated with imaging, physiological, and molecular data to simulate disease progression and treatment outcomes. This concept, originally applied in aerospace and manufacturing, is gaining momentum in biomedicine. In cardiovascular contexts, digital twins integrate high-resolution anatomical models derived from imaging with patient-specific biomarkers, risk factors, and sometimes omics profiles [129].

The digital twin functions as a dynamic computational model. For example, patient-derived coronary CT angiograms are used to reconstruct arterial geometry, while hemodynamic simulations predict fractional flow reserve (FFR) [130]. As longitudinal data, such as repeat imaging or blood biomarkers, are incorporated into the model, it can forecast plaque progression or response to therapy. Integration of omics data adds further personalization by predicting how an individual’s transcriptomic or proteomic signature may influence vascular remodeling under hypertensive stress [131,132].

The main limitations are the data intensity required and regulatory uncertainty. Creating accurate digital twins demands high-quality longitudinal datasets, which are seldom available in clinical practice [133]. The computational requirements are substantial, and models are only as reliable as the assumptions underlying them. Furthermore, digital twins are still considered investigational tools; their clinical utility must be demonstrated in prospective trials before widespread adoption. Issues related to data privacy and model interpretability also remain unresolved. 

### 6.6. Targeted Protein Degradation

PROTACs (proteolysis targeting chimeras) are innovative molecules that induce targeted protein degradation by recruiting E3 ubiquitin ligases to specific substrates. Unlike gene editing, which changes DNA sequences, PROTACs adjust protein levels directly. In 2020, a study reported the first PROTAC degrader targeting transmembrane HMGCR (3-Hydroxy-3-Methylglutaryl-CoA Reductase). This PROTAC overcomes statin resistance by inhibiting cholesterol biosynthesis without causing HMGCR upregulation [24].

## 7. Integration and Translational Outlook

### 7.1. scRNA-Seq Spatial Transcriptomics: Illuminating Gene Expression Within Vascular Architecture

Spatial transcriptomics (ST) marks a revolutionary advancement in transcriptomic profiling by preserving the spatial context of gene expression within intact tissue architecture. Unlike bulk or scRNA-seq, which disrupts tissue organization, ST preserves transcript localization, enabling researchers to visualize gene expression patterns alongside histological features. This approach is particularly transformative for cardiovascular and vascular disease studies, where the cellular microenvironment and spatial organization play crucial roles in disease pathogenesis [96,97,98].

In VDs such as atherosclerosis, aneurysms, and vascular remodeling, ST enables detailed mapping of transcriptional activity across specific regions within diseased tissue [99]. For example, a study using spatial transcriptomics to analyze atherosclerotic plaques revealed distinct zones of inflammation, necrosis, and fibrous tissue, each characterized by unique gene expression signatures linked to immune activation, extracellular matrix remodeling, and lipid metabolism. This level of resolution helps identify disease-driving cell types and interactions otherwise masked in dissociated single-cell analyses. ST clarifies the complex crosstalk between endothelial cells, vascular smooth muscle cells (VSMCs), fibroblasts, and immune cells in spatially confined regions by overlaying transcriptomic data on histological images [100]. For instance, macrophage subtypes enriched in pro-inflammatory cytokines can be mapped adjacent to lipid cores in plaques. At the same time, smooth muscle cell-derived foam cells may localize to fibrous cap regions, highlighting potential targets for intervention.

Spatial transcriptomics has also shown promise in characterizing aneurysmal walls, where gene signatures related to matrix degradation, angiogenesis, and immune cell infiltration are distributed heterogeneously. Understanding these spatial dynamics reveals mechanisms of disease progression and provides a basis for designing region-specific drug-delivery strategies or identifying tissue zones susceptible to rupture. Integrating ST with other omics technologies, such as scRNA-seq and proteomics, can further advance our understanding of vascular pathology. Such multimodal datasets support cross-validation of findings and help dissect the relationships among gene expression, protein localization, and functional changes in situ [98]. Additionally, the development of high-throughput spatial barcoding and advanced imaging platforms continues to enhance ST’s resolution and scalability, making it increasingly suitable for large-scale studies of human cardiovascular disease.

### 7.2. Multi-Omics Integration (scRNA-Seq, ATAC-Seq, and Proteomics)

**Figure 5:**
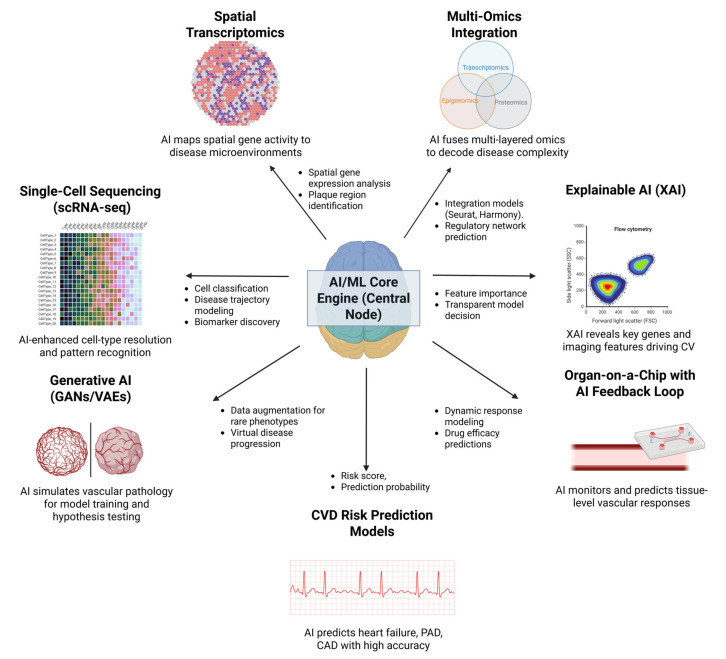
Integrative AI/ML framework for cardiovascular disease (CVD) modeling and prediction. This schematic illustrates the central role of AI/ML as a core engine for integrating diverse next-generation technologies to advance vascular disease research. Spatial transcriptomics enables mapping of gene activity within microenvironments for spatial gene expression analysis and plaque identification. ScRNA-seq provides AI-enhanced cell-type resolution for cell classification, disease trajectory modeling, and biomarker discovery. Multi-omics integration (including transcriptomics, proteomics, and epigenomics) leverages AI to fuse multi-layered data for regulatory network prediction and improved disease decoding. Generative AI (GANs/VAEs) simulates vascular pathology, supporting virtual disease progression modeling and data augmentation for rare phenotypes. CVD risk prediction models powered by AI accurately estimate heart failure, PAD, and CAD risk, yielding individualized risk scores. Explainable AI (XAI) enhances model transparency, revealing feature importance, key genes, and imaging drivers of cardiovascular phenotypes. Organ-on-a-chip systems, combined with AI feedback loops, enable monitoring of tissue-level vascular responses and predicting drug efficacy. This framework highlights how AI/ML-based integration of omics, computational modeling, and experimental systems can allow precision diagnostics, risk stratification, and therapeutic optimization in cardiovascular medicine. Abbreviations: AI/ML (artificial intelligence and machine learning), CVD (cardiovascular disease), scRNA-seq (single-cell RNA sequencing), PAD (peripheral artery disease), CAD (coronary artery disease), XAI (explainable AI), GAN (Generative adversarial networks), VAE (variational autoencoder). The figure was created in BioRender. Figure credit: Sahu, D. (2025) https://www.biorender.com/ (accessed on 12 November 2025).

Figure 5 presents a schematic of an AI/ML-centered framework integrating multi-omics, single-cell sequencing, spatial transcriptomics, generative AI, explainable AI, organ-on-a-chip systems, and risk prediction models to enhance cardiovascular disease modeling, prediction, and therapeutic optimization.

## 8. Clinical Translation and Regulatory Science

Several emerging technologies discussed in this review are already progressing from bench to bedside, with early-phase human studies and developing regulatory frameworks shaping their clinical adoption. In this section, we differentiate technologies that have already entered clinical workflows or early human studies from those that remain preclinical or exploratory, and we highlight key regulatory, validation, and implementation challenges specific to vascular disease.

### 8.1. Spatial Transcriptomics in Vascular and Oncologic Specimens

While spatial transcriptomics remains primarily a research tool, it has been successfully applied to resected human atherosclerotic plaques and to surgical vascular tissues. These studies have mapped region-specific immune, fibro-calcific, and angiogenic signatures, providing spatially anchored biomarkers that distinguish stable from rupture-prone plaques. Similar approaches have been incorporated into translational oncology trials, establishing methodological precedents and pipelines that can be adapted for cardiovascular research. The convergence of these efforts will accelerate biomarker discovery and may eventually support patient stratification in clinical trials of anti-inflammatory or plaque-stabilizing therapies [134,135]. Despite its power, spatial transcriptomics is currently restricted to research settings. No spatial transcriptomics-based assays are approved for clinical vascular diagnostics, and substantial barriers related to tissue handling, cost, standardization, and data interpretation must be addressed before clinical deployment.

### 8.2. Integration of Artificial Intelligence into Imaging Workflows

Machine learning tools for vessel segmentation, automated plaque burden quantification, and risk stratification are already being introduced into clinical practice. The U.S. Food and Drug Administration (FDA) maintains a growing list of AI-enabled imaging devices authorized for vascular and cardiovascular applications, and prospective validation studies are underway to assess their impact on diagnostic throughput and prognostic accuracy. These developments indicate that AI has moved beyond proof-of-concept to regulated clinical deployment in imaging-heavy fields relevant to vascular disease [136,137]. As these tools move from development to deployment, stringent external validation across institutions, imaging platforms, and patient demographics, along with continuous post-implementation performance monitoring, will be essential to ensure safety and mitigate bias.

### 8.3. Regulatory Science and Harmonization

Successful translation of these platforms requires alignment with evolving regulatory frameworks. The FDA has outlined a lifecycle approach for software as a medical device (SaMD), emphasizing good machine learning (GML) practices, predetermined change control plans, and post-market monitoring. The European Medicines Agency (EMA) has issued a work plan aligned with the forthcoming EU AI Act, emphasizing risk-based oversight, transparency, and traceability when AI informs patient care [138]. Parallel efforts by the U.S. National Institutes of Health (NIH), such as the Bridge2AI program, and international initiatives like the Human Cell Atlas provide community standards, ML-ready datasets, and metadata conventions that strengthen reproducibility and support regulatory acceptance [139]. Regulatory agencies increasingly emphasize not only analytical performance but also clinical utility, transparency of algorithms, and robust mechanisms for updating and revalidating models over time, particularly for adaptive AI-based devices.

### 8.4. Implications for Vascular Research

Several principles are emerging for investigators and developers. First, intended use and risk classification should be defined early, as regulatory expectations differ for decision-support versus interventional guidance. Second, both analytical validity (e.g., reproducibility, sensitivity, robustness to batch effects) and clinical utility (e.g., correlation with outcomes such as revascularization or event-free survival) should be demonstrated in multicenter studies. Third, continuous performance monitoring and harmonized metadata capture are increasingly non-negotiable requirements. By incorporating these considerations into experimental design, vascular technologies can advance beyond proof-of-concept to become validated tools with real clinical impact [140].

### 8.5. Emerging Hotspots in Vascular Disease Research

Several emerging research domains are poised to reshape how VDs are studied and managed. Multiscale modeling frameworks that couple hemodynamics, wall biomechanics, and biologically informed remodeling rules offer the potential to simulate disease trajectories and virtual interventions in silico [24]. Digital twins extend this concept to patient-specific virtual replicas that integrate anatomy, physiology, and, in some implementations, molecular or lifestyle data [133]. In parallel, high-dimensional proteomics and metabolomics are expanding the catalog of circulating and tissue-derived biomarkers beyond what can be captured by transcriptomics alone, while advances in nanotechnology and smart biomaterials are enabling increasingly sophisticated platforms for targeted drug delivery, molecular imaging, and local biomechanical modulation [141]. Epitranscriptomic profiling adds an additional regulatory layer by revealing how RNA modifications modulate vascular cell responses to inflammatory, mechanical, and metabolic cues [142].

Increasingly, these domains are being combined in integrated frameworks. Spatial omics and advanced imaging can be jointly analyzed to create multi-modal atlases that link microscopic cell states and molecular programs to macroscopic lesion phenotypes [143]. Multi-omics discovery pipelines are being coupled with organ-on-chip and organoid platforms to functionally test candidate pathways under physiologically relevant flow and cellular configurations [144]. Continuous physiological monitoring from wearables or implantable sensors is being explored as an input for digital twins and predictive models, enabling dynamic, patient-specific risk assessment and treatment adaptation [145]. These emerging combinations are largely at an exploratory or proof-of-concept stage and will require coordinated, multidisciplinary efforts to achieve clinical impact.

### 8.6. Proteomics and Metabolomics: Expanding Beyond Transcriptomics

Proteomics and metabolomics offer complementary perspectives to transcriptomics by directly measuring the abundance and modifications of proteins and metabolites, the functional molecules driving vascular physiology. Advances in high-resolution mass spectrometry (MS), tandem MS (LC–MS/MS), and targeted assays (ELISA, MRM panels) now enable systematic profiling of circulating proteins and metabolites in vascular disease cohorts [146,147].

Proteomic studies in vascular disease typically analyze plasma, serum, or tissue samples to identify dysregulated proteins associated with endothelial dysfunction, inflammation, and extracellular matrix remodeling [148]. For example, proteomic profiling has detected upregulation of osteogenic proteins (RUNX2, osteopontin) in vascular smooth muscle cells undergoing calcification. Metabolomics, by contrast, captures systemic metabolic shifts linked to vascular disease risk, such as elevated trimethylamine-N-oxide (TMAO) levels related to atherosclerosis or altered amino acid metabolism in hypertension. Integrating proteomics, metabolomics, and transcriptomics enables multi-omics network construction, revealing mechanistic pathways and identifying candidate biomarkers for risk stratification.

Several limitations hinder clinical translation. Proteomic data may be biased by sample handling, the dynamic protein range (where abundant proteins mask rare events), and inter-laboratory variability [149]. Metabolomic profiles are influenced by diet, microbiome, and medication, which complicates reproducibility [150]. Standardization of assay protocols, normalization strategies, and reference ranges is still in development. Large, prospective, multi-ethnic cohorts are needed to establish robust biomarker panels that provide predictive value beyond traditional risk factors.

To date, most proteomic and metabolomic signatures associated with vascular disease remain at the discovery or early validation stage, with limited multi-center replication and sparse evidence for incremental clinical value beyond established risk factors.

### 8.7. Nanotechnology and Smart Biomaterials

Nanotechnology has rapidly emerged as a tool for targeted drug delivery, vascular imaging, and tissue engineering. Liposomes, polymeric nanoparticles, and inorganic nanostructures can be functionalized with ligands to target diseased vascular sites, such as inflamed endothelium or lipid-rich plaques [151,152]. Innovative biomaterials, including bioresorbable scaffolds and stimuli-responsive stents, represent another frontier by enabling drug release or responding to local hemodynamic cues.

Nanoparticles exploit the enhanced permeability and retention (EPR) effect or ligand–receptor interactions, for example, by targeting vascular cell adhesion molecule-1 on inflamed endothelium, to localize selectively within vascular lesions [153]. They can deliver anti-inflammatory agents, siRNA, or imaging contrast agents. Smart stents and hydrogels can be engineered to release drugs in response to environmental triggers such as pH, reactive oxygen species, or shear stress [154,155]. The integration of these devices with sensors and wireless monitoring systems enables AI-guided feedback loops for vascular implants.

The main limitations involve safety, scalability, and regulatory hurdles. Nanoparticles can accumulate in non-target organs, such as the liver and spleen, which raises toxicity concerns. Long-term biocompatibility and the degradation products of novel biomaterials require rigorous evaluation. Manufacturing reproducibility at a clinical scale also remains a barrier, as does navigating the regulatory classification of combination products (drug + device + diagnostic) [156]. Despite promising preclinical data, these challenges have slowed clinical adoption. Although several nanotechnology-based formulations are clinically approved in oncology and other fields, vascular-specific nanotherapeutics and imaging agents are largely preclinical, and issues of long-term safety, biodistribution, manufacturing scalability, and regulatory classification remain major obstacles.

### 8.8. Epitranscriptomics: RNA Modifications as Regulators of Vascular Biology

Epitranscriptomics refers to chemical modifications of RNA molecules, such as N6-methyladenosine (m6A), pseudouridine, or 5-methylcytosine, that alter RNA stability, splicing, translation, or localization without changing the underlying sequence [157,158]. These modifications are mapped using high-throughput sequencing methods such as MeRIP-seq (m6A RNA immunoprecipitation sequencing), miCLIP (m6A crosslinking immunoprecipitation), or direct RNA sequencing with nanopore technologies [159].

m6A is the most abundant internal mRNA modification in eukaryotes and is dynamically regulated by “writers” (methyltransferases such as METTL3), “erasers” (demethylases like FTO), and “readers” (YTH domain proteins) [160,161]. In vascular biology, m6A influences endothelial cell proliferation, smooth muscle phenotypic switching, and inflammatory responses. For example, altered m6A modification can destabilize mRNAs encoding protective endothelial genes, exacerbating vascular injury [162]. Epitranscriptomic regulation thus represents a finely tuned mechanism that links environmental stressors to transcriptomic remodeling in vascular disease.

This field is nascent, with few studies directly linking RNA modifications to clinical vascular outcomes. Technical challenges include antibody specificity in immunoprecipitation assays, low resolution in mapping modifications, and variability across sequencing platforms [163,164]. Additionally, the causal role of RNA modifications, rather than merely correlative associations, remains to be clarified. Pharmacological targeting of epitranscriptomic regulators is an emerging, yet untested, strategy in cardiovascular disease.

### 8.9. Strengths and Limitations of These Assays

Strengths of single-cell and spatial omics include single-cell resolution, ability to uncover rare cell states and spatial niches, and power to generate mechanistic hypotheses about cell–cell communication. Limitations include high cost, complex workflows, sensitivity to tissue quality, batch effects, modest cohort sizes, and the fact that most derived biomarkers remain research-grade [165].

Imaging innovations include advanced modalities such as super-resolution microscopy, intravital imaging, photoacoustic imaging, and super-resolution ultrasound, which provide detailed structural and functional information beyond conventional imaging. However, trade-offs between resolution and penetration depth, technical complexity, and the need for specialized equipment and expertise limit their availability and standardization [166].

Table 4 summarizes the limitations of imaging modalities, along with representative depth and spatial resolution, and their current applications.

The above table compares the principal imaging modalities discussed in Section 4, focusing on their penetration depth, temporal resolution, and limitations in vascular disease imaging. It also summarizes the current preclinical and clinical readiness for each technique.

The microfluidic and organ-on-a-chip systems enable controlled manipulation of flow, shear stress, and cellular composition, thereby improving mechanistic modeling compared with static cultures. Their limitations include variability in device design, difficulty capturing full vascular heterogeneity and systemic interactions, long-term stability issues, and relatively low throughput [173].

CRISPR/Cas9 and optogenetic methods provide precise perturbations for the mechanistic dissection of gene function and cellular behavior. Concerns about off-target effects, delivery challenges, immunogenicity, and ethical and regulatory considerations constrain their clinical translation [174].

Machine learning, generative AI, multiscale modeling, and digital twins can integrate heterogeneous datasets, simulate complex vascular dynamics, and generate predictions at the patient level. Key limitations include data requirements, risk of bias, limited transparency and interpretability in some models, and uncertain performance when applied outside the training distribution [175]. To connect these emerging technologies to real-world clinical needs, Table 5 summarizes major vascular diseases, associated risk factors, diagnostic approaches, and current treatment strategies.

An overview of the current translational positioning of each major technology, ranging from discovery-stage mechanistic tools to emerging early clinical applications in vascular disease, is provided in Table 6. 

### 8.10. Strength of Evidence and Approach to Study Quality Assessment

The evidence base supporting emerging vascular imaging technologies is heterogeneous, with considerable variation in study design, sample size, technical maturity, and the level of independent validation [176]. Several modalities included in this review, such as photoacoustic imaging, super-resolution ultrasound, optogenetic vascular modulation, and microfluidic vascular models, are represented predominantly by feasibility studies or isolated reports conducted in limited preclinical settings [177]. In these areas, conclusions remain preliminary because protocols, acquisition parameters, and analytical workflows are not yet standardized across laboratories. Small datasets, single-center implementations, and unreplicated experimental configurations also contribute to uncertainty, making it difficult to determine the reproducibility and generalizability of reported effects [178].

Evidence is also inconsistent in domains where studies employ different imaging platforms, contrast agents, or biological models. Discrepancies in microvascular perfusion measurements, plaque-level characterization metrics, or endothelial signaling readouts often arise from variations in hardware sensitivity, reconstruction algorithms, or biological preparation rather than from true biological divergence [80]. We emphasized the need for larger, harmonized studies to clarify whether these observations reflect genuine physiological mechanisms or technology-dependent artifacts.

To assess the quality of each study, we examined multiple aspects, including the robustness of the experimental design and the clarity of the imaging methodology. We also considered whether the approach was validated against recognized reference standards. Studies that met these criteria and demonstrated consistent effects across multiple models or cohorts were given greater interpretive weight. Conversely, results derived from preliminary or single-study evidence were contextualized as early signals rather than established trends. This approach allowed us to distinguish robust, convergent findings from those that remain provisional and to identify areas where methodological refinement, multicenter validation, and standardized reporting would substantially strengthen the evidence base. Several advanced imaging modalities are now used to investigate vascular structure and function, yet each technology carries characteristic limitations that can influence data quality, interpretation, and reproducibility. Reports from preclinical and clinical studies describe depth-related signal loss, motion-sensitive acquisition artifacts, inconsistent hardware performance, and variability introduced by device design or analytical workflows [80]. These issues are particularly relevant in microvascular imaging, endothelial dynamics, plaque characterization, and vascular-on-chip platforms, where measurements depend heavily on spatial resolution, temporal stability, and standardized acquisition protocols [179]. To provide a clear overview, Table 7 below summarizes documented failure modes and reproducibility concerns for commonly used vascular imaging approaches, along with their specific applications and supporting primary references.

## 9. Conclusions and Future Prospects

VDs are driven by complex interactions among endothelial cells, vascular smooth muscle cells, fibroblasts, immune cells, and circulating factors within a dynamic hemodynamic environment. Emerging technologies reviewed here, including single-cell and spatial omics, advanced imaging, microfluidic and organ-on-chip systems, genetic perturbation tools, and AI-based computational frameworks, have substantially deepened our understanding of these processes. They have revealed previously unappreciated cellular heterogeneity, transitional cell states, and spatially confined microenvironments that contribute to atherosclerosis, aneurysm formation, pulmonary vascular remodeling, and microvascular dysfunction.

Some of these technologies are beginning to influence clinical practice. AI-supported imaging tools for vessel segmentation, plaque characterization, and risk prediction, as well as early photoacoustic imaging studies, are moving toward integration in vascular diagnostics. In contrast, most multi-omics platforms, microfluidic and organ-on-chip systems, CRISPR- and optogenetics-based perturbations, and digital twins remain in the preclinical or exploratory stages, where they primarily serve as engines for mechanistic discovery and hypothesis generation. Recognizing these different maturity levels is essential to set realistic expectations and prioritize translational investments.

Major bottlenecks in translation include technical complexity, cost, limited standardization, and the lack of large, diverse, multi-center datasets. For omics and imaging assays, reproducibility, protocol harmonization, robust quality control, and demonstration of incremental clinical utility over existing standards are critical. For organ-on-chip and genetic tools, issues of reproducibility, long-term stability, delivery, and safety need to be addressed. For AI and multiscale models, transparent reporting, external validation, fairness across populations, and explicit characterization of failure modes are essential prerequisites for regulatory approval and clinical trust.

Future efforts should prioritize the development of integrated pipelines that link discovery to application: multi-omics and spatial profiling to identify candidate mechanisms; AI and network analysis to prioritize and model these mechanisms; physiologically relevant microfluidic and organ-on-chip systems to test interventions; and multiscale models and digital twins to translate insights to patient-specific prediction and decision support. Multi-center consortia, shared reference datasets, and close collaboration between clinicians, biologists, engineers, and regulatory agencies will be indispensable. If these challenges are addressed, the technologies described here have the potential to enable more precise diagnostics, targeted therapies, and ultimately, personalized vascular medicine.

## Figures and Tables

**Table 1:** Applications of optogenetics in cardiovascular research across different cell types and disease models. The table summarizes current uses of optogenetics in VSMCs, ECs, and combined EC–VSMC systems, as well as in general CVD models. For each category, key cardiovascular diseases studied, mechanisms and outcomes explored, major challenges, and potential future directions are highlighted. This overview underscores how optogenetics provides a platform for dissecting cell-specific and intercellular mechanisms underlying vascular dysfunction and remodeling, while also highlighting the technical hurdles and translational opportunities for advancing this field. Abbreviations: CRISPR (clustered regularly interspaced palindromic repeats), CVD (cardiovascular diseases), EC (endothelial cells), VSMC (vascular smooth muscle cells).

Cell Type	Optogenetic Application	Relevant Cardiovascular Disease(s)	Mechanisms/Outcomes	Challenges	Future Directions
VSMCs	Modulation of contractility and phenotype switching	Hypertension, Atherosclerosis, Restenosis, Arteriosclerosis	Control of vascular tone (e.g., inducing contraction) Study of phenotypic switching between contractile and synthetic states	Light penetration in thick tissues Efficient opsin delivery to specific VSMC regions	Development of more tissue-penetrating opsins Application of optogenetics in patient-derived models to study disease progression
ECs	Modulation of nitric oxide (NO) production under disturbed flow	Atherosclerosis, Thrombosis, Vascular Remodeling	Study of endothelial dysfunction Control of NO synthesis and endothelial permeability Modulation of endothelial cell behavior in response to shear stress	Limited depth of light penetration Difficulty in achieving precise control of endothelial functions in vivo	Combining optogenetics with flow dynamics studies Realtime monitoring of endothelial response in disease models
VSMCs and ECs Combined	Intercellular communication under flow and stress conditions	Atherosclerosis, Hypertension, Heart Failure	Investigation of EC-VSMC signaling Study of paracrine signaling between cells Control of vascular remodeling responses	Complexity of simultaneous modulation of two cell types Need for synchronized light activation in vivo	Optogenetic interventions targeting both ECs and VSMCs to prevent or reverse atherosclerosis and plaque rupture
General Cardiovascular Models	Optogenetics in animal models (in vivo manipulation)	General CVD (Hypertension, Atherosclerosis, Ischemia)	Real-time control of cellular activity, Assessment of therapeutic interventions in dynamic cardiovascular environments	In vivo implementation challenges: Immune responses to opsins or viral vectors	Refinement of in vivo optogenetic techniques, Integration of optogenetics with other therapies (e.g., CRISPR, gene therapy) for personalized medicine

**Table 2:** Gene and microRNA targets explored in cardiovascular disease using functional studies and genome-editing approaches. The table summarizes disease contexts, mechanistic pathways, and outcomes associated with specific targets, including miRNAs (miR-126, miR-145, miR-21, miR-155, miR-33), cholesterol transporters (ABCA1), transcriptional regulators (PBX1), CRISPR/Cas9 components, and regulatory enhancers. These studies highlight how genetic and epigenetic modulation informs vascular biology, lipid metabolism, immune regulation, and cardiac development, while also demonstrating the translational potential of gene- and miRNA-based interventions in cardiovascular disease. Abbreviations: ABCA1 (ATP-binding cassette transporter A1), Cas-9 (CRISPR-associated protein 9), CHD (coronary heart disease), CRISPR (clustered regularly interspaced palindromic repeats), EC (endothelial cells), HDL (high density lipoprotein), miR (microRNA), PBX1 (pre-B-cell leukemia transcription factor 1), VSMC (vascular smooth muscle cells).

Gene/miRNA Targeted	Disease Context	Mechanistic Insight/Target Pathway	Outcome
miR-126	Atherosclerosis	Vascular homeostasis, endothelial repair	Impaired angiogenesis, increased plaque formation
miR-145	Atherosclerosis	VSMC phenotypic switching (contractile → synthetic)	Plaque expansion upon deletion
miR-21	Atherosclerosis	Inflammation, endothelial function	Reduced plaque size; improved vascular tone
miR-155	Atherosclerosis	Immune modulation, endothelial dysfunction	Reduced inflammation, enhanced endothelial function
miR-33	Atherosclerosis	Cholesterol homeostasis (*ABCA1*/*ABCG1* regulation)	Improved lipid metabolism; reduced plaque burden
*ABCA1*	Atherosclerosis	Cholesterol efflux (HDL metabolism)	Validated its role in lipid regulation and atheroprotection
*PBX1* (missense variant)	Congenital Heart Disease	Cardiac development gene regulatory network	Functional effect of novel variant validated
Cas9 (expression validation)	Cardiovascular gene editing	CRISPR/Cas9 platform safety	Cas9 expression had no adverse effect on cardiac function
Unspecified enhancer	Coronary Heart Disease	Enhancer function in regulating CHD-related genes	Regulatory elements functionally mapped using CRISPR interference
miR-126/miR-221 co-edit	Vascular remodeling	Proliferation vs. quiescence balance in endothelial cells	Altered wound repair, proliferation kinetics

**Table 3:** Emerging techniques in cardiovascular research and their translational potential. The table outlines the key features, applications, strengths, and limitations of scRNA-seq, optogenetics, super-resolution microscopy, microfluidic devices, machine learning, and CRISPR/Cas9 gene editing. Together, these approaches enable high-resolution mapping of cellular mechanisms, functional interrogation of vascular and cardiac processes, and development of predictive and therapeutic strategies. The comparison highlights the complementary nature of these technologies while underscoring current technical, analytical, and ethical challenges. Abbreviations: CRISPR (clustered regularly interspaced palindromic repeats), Cas-9 (CRISPR-associated protein 9), miRNA (microRNA), scRNA-seq (single-cell RNA sequencing), VSMC (vascular smooth muscle cells).

Technique	Key Features	Applications	Strengths	Limitations
scRNA-seq	Cell-level transcriptomics	Cell heterogeneity, biomarker discovery	Cell-specific insights, disease subtype profiling	High cost, data complexity, complex analysis
Optogenetics	Light-activated gene/protein control	VSMC activity study, cardiac-neural interactions	Precision control of cell activity	Poor tissue penetration, limited in vivo application
Super-resolution Microscopy	Imaging beyond diffraction limit	Microvascular and plaque imaging	Ultra-high spatial resolution	Requires expertise and advanced, costly equipment
Microfluidic Devices	Lab-on-chip vascular modeling	Shear stress studies, thrombosis models	Real-time simulation of blood flow	Limited physiological mimicry, scalability issues
Machine Learning	Data-driven pattern recognition	Risk prediction, precision diagnostics	High predictive accuracy, real-world clinical use	Data quality dependency, lack of interpretability
CRISPR/Cas9 Gene Editing	Targeted gene manipulation	Gene function study, miRNA targeting	Specific genetic targeting, therapeutic potential	Off-target effects, ethical concerns, delivery limitations

**Table 4:** Comparative analysis of imaging modalities investigated in vascular diseases. Abbreviations: Hb (hemoglobin), IVM (intravital microscopy), PAI (photoacoustic imaging), PAT (photoacoustic tomography), SIM (structured illumination microscopy), ULM (ultrasound localization microscopy), uULM (ultrafast ultrasound localization microscopy), X-Ray (X-ray), µm (micrometer).

Imaging Modality	Penetration Depth in Tissue	Resolution (Spatial/Axial/Temporal)	Key Limitations for Vascular Disease Work	Current Readiness	References
Super-resolution optical microscopy (2-photon/SIM-type approach demonstrated in heart tissue)	70 µm deep in the mouse heart muscles	150 nm spatial resolution	Limited resolution caused by optical aberrations and scattering from dense biological samples.	Preclinical research stage in mouse heart muscles	[167]
PAI/PAT	3–4 mm depth in beating heart of mouse	Axial and lateral resolutions 27.7 and 3.6 μm	Penetration depth is restricted by optical and acoustic attenuation, and the lack of an endogenous PA signal from Hb, which limits early thrombosis detection.	Used at preclinical research	[168]
Super-resolution ultrasound ULM/uULM	30 and 120 mm	1700 and 5850 μm spatial resolution	Currently processed as offline	Preclinical research	[169]
Synchrotron Radiation X-Ray Phase-Contrast Tomography	0.095 to 0.302 mm	~3.7 μm	Enhancing imaging contrast of vasculature is challenging because the X-ray wavefronts refracted at each interface between blood flow in the vessel lumen and the surrounding tissue are difficult to distinguish in both vivo and in vitro settings.	Preclinical research	[170,171]
IVM, two-photon IVM	Two-photon intravital reports kidney depth of 150–200 µm and brain > 1 mm	Optic resolution around 250 nm;	Limited laser penetration into the tissue; quantitative analysis begins with image acquisition.	Preclinical studies	[172]

**Table 5:** Overview of major vascular diseases, pathology, prevalence, symptoms, complications, risk factors, diagnostic approaches, and treatment strategies. The table includes atherosclerosis, peripheral artery disease (PAD), deep vein thrombosis (DVT), varicose veins, and aneurysms, outlining how these conditions contribute to the global cardiovascular disease burden. It highlights the shared and distinct features of disorders, underscoring the importance of early diagnosis, risk factor management, and tailored therapeutic interventions. Abbreviations: ABI (ankle-brachial index), CT (computed tomography), CVD (cardiovascular diseases), DVT (deep vein thrombosis), MRI (magnetic resonance imaging).

Disease	Pathology	Prevalence	Symptoms	Complications	Key Risk Factors	Diagnostic Tools	Treatment Options
Atherosclerosis	Cholesterol and plaque buildup in arteries	Global; major CVD contributor	Chest pain, fatigue, shortness of breath	Heart attack, stroke, angina	Smoking, high cholesterol, hypertension, diabetes, and age	Angiography, CT, blood lipids, ultrasound	Statins, antihypertensives, stents, lifestyle
Peripheral Artery Disease	Arterial narrowing, mostly in the lower limbs	>200 million globally	Calf/thigh pain during walking (claudication)	Amputation, stroke, limb ischemia	Smoking, diabetes, age, obesity, and hypertension	Ankle-Brachial Index (ABI), Doppler ultrasound	Antiplatelets, statins, angioplasty, exercise
Deep Vein Thrombosis (DVT)	Clot formation in deep veins (legs, pelvis)	Common in immobile/post-surgical patients	Leg swelling, pain, redness	Pulmonary embolism, post-thrombotic syndrome	Immobility, surgery, cancer, pregnancy, and coagulation disorders	D-dimer test, venous Doppler ultrasound	Anticoagulants, compression stockings, thrombolysis
Varicose Veins	Dysfunctional valves leading to vein dilation	Affects ~25–33% women, 20% men	Aching legs, visible bulging veins	Ulcers, bleeding, thrombophlebitis	Standing jobs, obesity, pregnancy, age, and heredity	Duplex ultrasound, physical exam	Compression therapy, laser ablation, vein stripping
Aneurysm	Weakening and bulging of the vessel wall	Abdominal aneurysm: ~2–8% in men over 65	Often asymptomatic until rupture	Rupture, internal bleeding, sudden death	Smoking, atherosclerosis, hypertension, genetics	CT angiography, ultrasound, MRI	Monitoring, surgical clipping, and endovascular repair

**Table 6:** Translational stage of emerging technologies in vascular disease. The above table summarizes the approximate translational stages of key emerging technologies discussed in this review, grouped by modality. For each technology category, representative examples, predominant vascular applications, and the current position along the discovery–preclinical–pilot human–clinical continuum are indicated. Stages reflect predominant use in vascular disease at the time of writing and are not intended as formal regulatory classifications. Abbreviations: AI (artificial intelligence), ATAC-seq (assay for transposase-accessible chromatin using sequencing), CE (Conformité Européenne; CE marking), ChIP-seq (chromatin immunoprecipitation sequencing), CRISPR (clustered regularly interspaced palindromic repeats), CRISPRa (CRISPR activation), CRISPRi (CRISPR interference), CT (computed tomography), FDA (Food and Drug Administration), GAN (generative adversarial network), m6A-seq (N6-methyladenosine sequencing), MeRIP-seq (m6A RNA immunoprecipitation sequencing), ML (machine learning), MR (magnetic resonance), MRI (magnetic resonance imaging), MSOT (multispectral optoacoustic tomography), PALM (photoactivated localization microscopy), scATAC-seq (single-cell ATAC sequencing), scRNA-seq (single-cell RNA sequencing), SIM (structured illumination microscopy), STED (stimulated emission depletion microscopy), VAE (variational autoencoder), XAI (explainable artificial intelligence).

Technology Category	Representative Examples	Predominant Vascular Application	Current Translational Stage
Single-cell and spatial omics	scRNA-seq, scATAC-seq, spatial transcriptomics	Mapping cellular heterogeneity, cell–cell communication, and spatial niches in atherosclerotic plaques, aneurysms, and pulmonary vascular remodeling	Discovery
Epigenetic and chromatin accessibility assays	DNA methylation profiling, ChIP-seq, single-cell ATAC-seq	Identifying regulatory elements, transcription factor networks, and epigenetic remodeling in endothelial cells, vascular smooth muscle cells, and lesional immune cells	Discovery
Proteomics and metabolomics	Mass spectrometry–based proteomics, imaging mass cytometry, untargeted/targeted metabolomics	Discovery of tissue and circulating protein and metabolite signatures associated with plaque instability, aneurysm progression, microvascular dysfunction, and treatment response	Discovery/early validation
Super-resolution and intravital optical imaging	STED, SIM, PALM/STORM, intravital microscopy	Mechanistic visualization of endothelial junctions, leukocyte–endothelium interactions, platelet adhesion, and microvascular dynamics in preclinical models	Preclinical
Photoacoustic imaging and tomography	Multispectral optoacoustic tomography (MSOT), volumetric photoacoustic tomography	Assessment of perfusion, oxygenation, and microvascular remodeling in peripheral artery disease and other vascular conditions	Pilot human
Advanced and super-resolution ultrasound	Ultrasound localization microscopy, contrast-enhanced super-resolution ultrasound	High-resolution mapping of microvascular structure and flow in deeper vascular beds, including early human studies	Preclinical/pilot human
Microfluidic and organ-on-a-chip systems	Vascular-on-a-chip, thrombosis-on-chip, patient-derived microvascular chips	Modeling hemodynamics, endothelial dysfunction, thrombosis, and drug responses under controlled flow using human or patient-derived cells	Preclinical
CRISPR/Cas9 and genome editing tools	CRISPR/Cas9 knockout/knock-in, base editing, CRISPRi/CRISPRa	Mechanistic dissection of gene function in endothelial cells, vascular smooth muscle cells, and immune cells; modeling monogenic vascular disorders	Preclinical (no approved therapies for vascular indications)
Optogenetic modulation of vascular/autonomic function	Channelrhodopsin-based modulation of sympathetic nerves or vascular smooth muscle cells	Experimental control of vascular tone, cardiac autonomic activity, and microcirculatory function in animal models	Preclinical/experimental
Discriminative AI and explainable ML	Deep learning for vessel segmentation, plaque characterization, risk prediction with XAI	Automated analysis of CT, MR, and ultrasound angiography; prediction of vascular events and treatment outcomes, with interpretability methods supporting mechanistic insight and clinician trust	Early clinical (some FDA/CE-marked tools, prospective studies)
Generative AI and synthetic data models	Variational autoencoders, generative adversarial networks	Data augmentation, simulation of vascular disease progression, and generation of synthetic vascular images or omics profiles for model development	Discovery/preclinical
Multiscale modeling	Coupled fluid–structure interaction models, growth and remodeling models	Simulation of hemodynamics, wall stress, and structural remodeling at patient- or cohort-level, with potential to support planning of vascular interventions	Preclinical/pilot human
Digital twins of the vascular system	Patient-specific virtual replicas integrating anatomy, physiology, longitudinal data	In silico testing of interventions, prediction of disease trajectories, and individualized risk assessment	Preclinical/pilot human (experimental)
Nanotechnology and smart biomaterials	Targeted nanoparticles, drug-eluting stents/grafts with responsive coatings, theranostic nanocarriers	Targeted delivery of drugs, genes, or imaging agents to vascular lesions; modulation of local biomechanical and inflammatory environments	Preclinical for vascular-specific applications (some related formulations approved in other fields)
Epitranscriptomics and RNA modification profiling	m6A-seq, MeRIP-seq, direct RNA sequencing for RNA modifications	Early-stage mapping of RNA modifications that regulate vascular cell responses to shear stress, hypoxia, metabolic and inflammatory stimuli	Discovery

**Table 7:** Technical limitations, failure modes, and reproducibility concerns with commonly used vascular imaging modalities. Each modality is listed with its primary vascular application, the specific issue identified in published studies, and the corresponding source. Abbreviations: IVUS (intravascular ultrasound), PAI (photoacoustic imaging), ULM (ultrasound localization microscopy).

Vascular Application/Context	Imaging Modality	Limitations/Failures	References
Superficial vasculature and minimally invasive vascular procedures (general PAI applications)	Conventional non-invasive photoacoustic imaging (PAI)	Strong optical attenuation limits PAI to imaging tissues only within a few centimeters of depth.	[180]
In-human hepatic and renal microvasculature	Ultrasound Localization Microscopy (ULM)	Insufficient sampling of the ULM system’s point-spread function leads to aliasing artifacts, compromising both microbubble localization and motion correction accuracy.	[181]
In-human microvascular imaging (capillary-scale vasculature)	ULM	The inability to fully resolve the capillary bed remains a key limitation of in-human ULM.	[86]
In vivo cardiac microcirculation (beating heart)	Intravital fluorescence microscopy	Tissue displacement induced by cardiac and respiratory activity significantly restricts the application of intravital fluorescence microscopy.	[182]
In vivo imaging of moving organs (including heart vasculature)	Intravital microscopy (laser scanning)	Intravital microscopy continues to face major obstacles from motion-induced artifacts during in vivo organ imaging.	[182]
Coronary artery stenosis assessment	Conventional catheter-based coronary angiography	Conventional coronary artery stenosis assessment does not capture the hemodynamic impact of stenoses or accurately characterize subendothelial structural features.	[183]
Vulnerable coronary plaque imaging	Intravascular ultrasound (IVUS)	The performance of IVUS is constrained by moderate resolution along with image artifacts and noise.	[184]
Carotid artery stenosis grading and plaque assessment	Carotid duplex ultrasound (velocity criteria)	Carotid duplex ultrasound relies on velocity-based criteria for stenosis grading; however, the absence of universally accepted consensus standards leads to variability in interpretation and clinical decision-making.	[185,186]

## Data Availability

No new data were created or analyzed in this study. Data sharing is not applicable.

## Abbreviations

AAV	Adeno-Associated Virus
ABC	ATP-Binding Cassette
ABCA1	ATP-binding cassette transporter A1 (gene/protein symbol)
ABCA1/ABCG1	ABCA1 and ABCG1 (cholesterol transporter genes)
ABI	Ankle–Brachial Index
AI	Artificial Intelligence
AI/ML	Artificial intelligence/machine learning
ApoE	Apolipoprotein E
ArchT	Archaerhodopsin-T
ATAC-seq	Assay for Transposase-Accessible Chromatin using sequencing
BBB	Blood–Brain Barrier
Bridge2AI	Bridge2AI (NIH data-generation initiative; proper name used in text)
C1QC	Complement C1q C chain (gene/protein symbol)
CAD	Coronary Artery Disease
Cas9	CRISPR-associated protein 9
CASP3	Caspase-3 (gene/protein symbol)
CD28null	CD28-null (CD28−) T-cell phenotype
CD4+	CD4-positive (cell-surface marker)
CDKN2A	Cyclin-dependent kinase inhibitor 2A (gene/protein symbol)
CE	Conformité Européenne (CE marking)
CFD	Computational fluid dynamics
CHD	Coronary heart disease
ChR2	Channelrhodopsin-2
ChrimsonR	Red-shifted Channelrhodopsin Variant
CITE-seq	Cellular Indexing of Transcriptomes and Epitopes by sequencing
CMD	Coronary Microvascular Dysfunction
COL1A1	Collagen type I alpha 1 chain (gene/protein symbol)
CRISPR	Clustered Regularly Interspaced Short Palindromic Repeats
CRISPR-Cas9	CRISPR–Cas9 gene-editing system
CRNN	Convolutional Recurrent Neural Network
CSN	Cardiac Sympathetic Nerve
CT	Computed Tomography
CVD	Cardiovascular Disease
CVDs	Cardiovascular diseases
dT	deoxythymidine (as in oligo(dT) primer)
DNA	Deoxyribonucleic acid
DVT	Deep vein thrombosis
EC	Endothelial Cell
ELISA	Enzyme-linked immunosorbent assay
EMA	European Medicines Agency
ERM	Empirical Risk Minimization
ETF-1	Essential Transcription Factor-1
EU	European Union
FDA	Food and Drug Administration
FFR	Fractional flow reserve
FTO	FTO (fat mass and obesity-associated protein; m6A demethylase)
GAN	Generative Adversarial Network
GML	Guided machine learning
GWAS	Genome-Wide Association Studies
HDL	High-density lipoprotein
HFpEF	Heart Failure with Preserved Ejection Fraction
HMGCR	3-Hydroxy-3-Methylglutaryl-CoA Reductase
ICMJE	International Committee of Medical Journal Editors
IGF1	Insulin-like growth factor 1 (gene/protein symbol)
iPSC	Induced Pluripotent Stem Cell
IVM	Intravital Microscopy
IVUS	Intravascular Ultrasound
KARS	Lysyl-tRNA synthetase (KARS; gene/protein symbol)
L	liter
LC-MS/MS	Liquid chromatography–tandem mass spectrometry
LDLR	Low-density lipoprotein receptor (gene/protein symbol)
LIME	Local Interpretable Model-agnostic Explanations
MeRIP-seq	m6A RNA immunoprecipitation sequencing
METTL3	Methyltransferase-like 3 (m6A ‘writer’; gene/protein symbol)
MI	Myocardial Infarction
miR-126	microRNA-126
miR-145	microRNA-145
miR-155	microRNA-155
miR-21	microRNA-21
miR-33	microRNA-33
miRNA	microRNA(s)
ML	Machine Learning
mm	millimeter
mRNA	messenger RNA
MRM	Multiple reaction monitoring
MRI	Magnetic Resonance Imaging
MS	Mass Spectrometry
MSOT	Multispectral Optoacoustic Tomography
MUSM	Multimodal Ultrafast Sonography Microscopy
MVD	Microvascular dysfunction
Myh11	Myosin heavy chain 11 (gene/protein symbol; mouse/vascular marker)
N6A	N6-adenosine (context: m6A modification)
N6C	N6-cytidine (as written; context-dependent)
NIH	National Institutes of Health
nm	nanometer
NO	Nitric oxide
NpHR	Natronomonas pharaonis Halorhodopsin
OoC	Organ-on-Chip
PAD	Peripheral arterial disease
PAH	Pulmonary Arterial Hypertension
PAI	Photoacoustic Imaging
PALM/STORM	Photoactivated localization microscopy/stochastic optical reconstruction microscopy
PA	Pulmonary Artery
PAT	Photoacoustic Tomography
PBX1	Pre-B-cell leukemia transcription factor 1 (gene/protein symbol)
PCR	Polymerase chain reaction
PCSK9	Proprotein convertase subtilisin/kexin type 9 (gene/protein symbol)
PROTAC	Proteolysis Targeting Chimera
PubMed	PubMed (biomedical literature database; proper name used in text)
RNA	Ribonucleic acid
RNA-seq	RNA sequencing
RUNX2	Runt-related transcription factor 2 (gene/protein symbol)
SaMD	Software as a medical device
scATAC-seq	Single-cell ATAC-seq (chromatin accessibility sequencing)
scRNA-seq	Single-cell RNA Sequencing
SHAP	Shapley Additive exPlanations
SIM	Structured illumination microscopy
s	second
SRM	Super-Resolution Microscopy
SRU	Super-Resolution Ultrasound
SRµT	Synchrotron Radiation Micro-Tomography
ST	Spatial transcriptomics
STED	Stimulated emission depletion (microscopy)
Tie2	Tie2 (TEK receptor tyrosine kinase; angiopoietin receptor)
TMAO	Trimethylamine N-oxide
TREM2-SPP1+	TREM2–SPP1 positive (macrophage phenotype marker set)
ULM	Ultrasound Localization Microscopy
ULM/uULM	Ultrasound localization microscopy/ultrafast ULM (as used in text)
VAE	Variational Autoencoder
VD	Vascular Disease
VSMC	Vascular Smooth Muscle Cell
WHO	World Health Organization
X-Ray	X-ray
XAI	Explainable Artificial Intelligence
YTH	YTH domain (YT521-B homology; m6A reader proteins)
µCCCM	Microfluidic Cardiac Cell Culture Models
µm	micrometer (micrometre)
